# *Peg3* Mutational Effects on Reproduction and Placenta-Specific Gene Families

**DOI:** 10.1371/journal.pone.0083359

**Published:** 2013-12-31

**Authors:** Joomyeong Kim, Wesley D. Frey, Hongzhi He, Hana Kim, Muhammad B. Ekram, Arundhati Bakshi, Mohammad Faisal, Bambarendage P. U. Perera, An Ye, Ryoichi Teruyama

**Affiliations:** Department of Biological Sciences, Louisiana State University, Baton Rouge, Louisiana, United States of America; University of Bonn, Institut of experimental hematology and transfusion medicine, Germany

## Abstract

*Peg3* (paternally expressed gene 3) is an imprinted gene encoding a DNA-binding protein. This gene plays important roles in controlling fetal growth rates and nurturing behaviors. In the current study, a new mutant mouse model has been generated to further characterize the functions of this DNA-binding protein. Besides known phenotypes, this new mutant model also revealed potential roles of *Peg3* in mammalian reproduction. Female heterozygotes produce a much smaller number of mature oocytes than the wild-type littermates, resulting in reduced litter sizes. According to genome-wide expression analyses, several placenta-specific gene families are de-repressed in the brain of *Peg3* heterozygous embryos, including prolactin, cathepsin and carcinoembryonic antigen cell adhesion molecule (Ceacam) families. The observed de-repression is more pronounced in females than in males. The de-repression of several members of these gene families is observed even in the adult brain, suggesting potential defects in epigenetic setting of the placenta-specific gene families in the *Peg3* mutants. Overall, these results indicate that *Peg3* likely controls the transcription of several placenta-specific gene families, and further suggest that this predicted transcriptional control by *Peg3* might be mediated through unknown epigenetic mechanisms.

## Introduction

*Peg3* (paternally expressed gene 3) is an imprinted gene located in proximal mouse chromosome 7/human chromosome 19q13.4 [1]–[3]. The genomic interval surrounding this gene is filled with lineage-specific Kruppel-type zinc finger genes [3]. *Peg3* itself is thought to have been derived from this type of ZNFs based on the presence of 12 zinc finger motifs within its ORF (Open Reading Frame) [1]–[3]. *Peg3* is placenta mammal-specific: the homologues are found only within eutherian mammals, but not in other mammals, such as metatherian and monotremes [4]. According to earlier studies on a mutant mouse line targeting *Peg3*, this gene is involved in controlling milk ‘letdown’ processes and nurturing behaviors in females [5],[6]. Later studies demonstrated that this gene also controls adipocyte differentiation and sexual behaviors in males [7],[8]. The imprinting of *Peg3* is regulated through a 4-kb CpG island surrounding its 1^st^ exon, termed the Peg3-DMR (Differentially Methylated Region) [9]. The Peg3-DMR contains multiple YY1 binding sites [10]. YY1 has subsequently been implicated in establishment and/or maintenance of the allele-specific DNA methylation at the Peg3-DMR [11]–[13]. In humans, the PEG3-DMR is known to be an epigenetically susceptible region: many patients of ovarian, breast and glioma cancers tend to lose the expression of *PEG3* mainly due to DNA hypermethylation on the PEG3-DMR [14]–[19]. Interestingly, reintroducing *PEG3* into primary cell lines derived from these cancer patients inhibited cell growth, demonstrating tumor suppressor activity [17],[19]. Thus, *PEG3* has been often regarded as a potential tumor suppressor in humans.

PEG3 is known to interact with several key proteins for various cellular processes. First, PEG3 interacts with TRAF2 (TNF receptor-associated factor 2), which controls the TNF (tumor necrosis factor) responsive pathway by activating NF-kB (NF-kappa-B) [20]. Second, PEG3 also interacts with SIAH1A (E3 ubiquitin-protein ligase SIAH1A), and this interaction triggers the induction of p53-mediated apoptosis in various cell lineages [21]. Consistent with this, several environmental insults, including hypoxic conditions, are known to up-regulate both PEG3 and p53 in cell lines as well as animal models, suggesting that *Peg3* may be a downstream gene for the p53-dependent apoptosis pathway [22]. Third, PEG3 is also known to inhibit the Wnt signaling pathway by promoting β-catenin degradation [23]. Lately, *Peg3* (also known as *Pw1*) has been recognized as a gene marker for myogenic progenitor cells based on the demonstration that a population of PW1-positive cells can regenerate skeletal muscle [24],[25]. Besides these various roles at the cellular level, recent studies using ChIP (Chromatin ImmunoPrecipitation) also revealed potential protein functions of PEG3 [26]. According to the results, PEG3 binds to specific genomic regions as a DNA-binding protein. Subsequently, many genomic loci have been identified as potential downstream targets for PEG3. Follow-up studies further demonstrated that PEG3 functions as a repressor in the transcription of these potential downstream genes [26].

Despite earlier studies, there are still many knowledge gaps between the known functions of *Peg3* at different levels. For instance, although PEG3 is regarded as a DNA-binding protein at the molecular level, it is unclear how this DNA-binding protein is involved in controlling animal behaviors and growth rates at the organismal level. To further characterize the functions of *Peg3*, we have generated a new mutant mouse model in the current study. This new model has been used for performing a series of breeding and expression analyses. The results indicate that *Peg3* may play important roles in mammalian reproduction. Also, genome-wide expression analyses indicate that several placenta-specific gene families are de-repressed in the brain of Peg3-heterozygous embryos and adults. This study further suggests that *Peg3* is involved in the transcriptional control of these placenta-specific gene families.

## Results

### Generation of a new mutant allele

**Figure 1:**
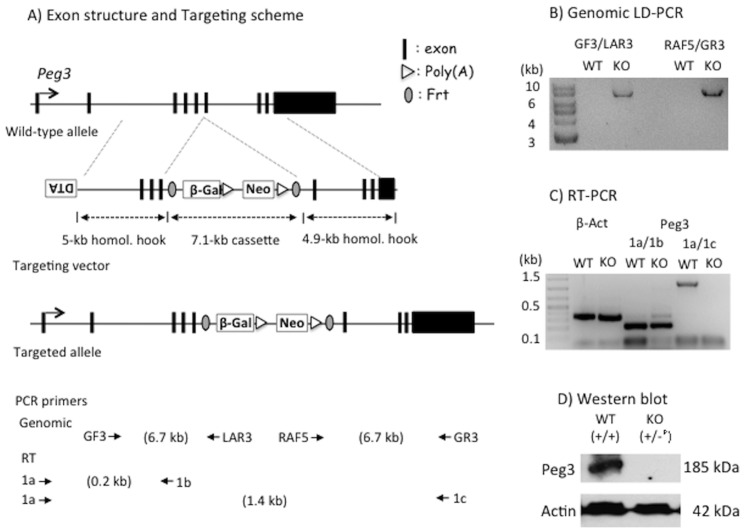
Exon structure and targeting scheme of the *Peg3* locus. (**A**) Schematic representations of the genomic locus of mouse *Peg3* and the targeting vector. The 9 exons of *Peg3* are indicated with vertical lines. The targeting vector is made of a negative selection marker (DTA, Diphtheria Toxin A), an insertion cassette and two homology hooks. The relative positions of two homology hooks are shown along with the genomic structure of *Peg3* with dotted lines. The 7.1-kb insertion cassette contains promoterless galactosidase (β-Gal) and human β-actin promoter-driven neomycin resistance gene (Neo). The bottom panel shows the relative positions of PCR primers with small arrows and the predicted sizes of genomic and RT-PCR products. (**B**) Genomic LD (Long Distance)-PCR. Two different sets of primers successfully amplified 6.7-kb genomic fragments from the genomic DNA of a heterozygote animal (KO), but not from that of a wild-type littermate (WT), confirming the proper targeting of the insertion cassette. (**C**) RT-PCR. RT-PCR was performed using the total RNA from WT and KO animals. The two primer sets (β-actin and 1a/1b) amplified similar levels of products between KO and WT, but the third primer set (1a/1c) flanking the insertion cassette amplified only from WT, confirming the proper truncation of *Peg3* transcription in KO. (**D**) Western blot analyses using the protein extracts prepared from the neonatal brains of WT and KO. This analysis revealed the absence of PEG3 protein in the pup inheriting the KO allele paternally.

A new mutant allele for *Peg3* was generated using a targeted ES cell (Peg3^tm1a(EUCOMM)hmgu^), which was obtained from the EUCOMM (European Conditional Mouse Mutagenesis program) [27]. In this ES clone, the 5^th^ intron of *Peg3* has been targeted by the insertion of a 7.1-kb cassette containing the promoterless *LacZ* gene and the neomycin resistance gene driven by the human β-actin promoter (**Fig. 1A**). This cassette was inserted through homologous recombination using two hooks: a 5.0-kb genomic region encompassing exon 3–5 and a 4.9-kb genomic fragment encompassing exons 6–8 and part of exon 9. The proper targeting was confirmed by long-distance PCR (**Fig. 1B**). The targeted ES clone (C57BL/6-derived JM8.N4 with the C/C genotype, black coat color) was injected into blastocysts that were harvested from albino females (C57BL/6 with the c/c genotype). This injection produced five chimeras with varying degrees of black coat color contribution ranging from 60 to 100%. Out of these five chimeras, two were successful in breeding with C57BL/6N females, generating 14 F1 mice out of 20 litters with germline transmission of the targeted allele. However, only 6 pups (2 males and 4 females) survived to the adulthood whereas the remaining 8 pups were dead on their first day.

The high level of perinatal lethality at the F1 generation was expected, since the gene dosage of *Peg3* is already null among the F1 pups with the target allele. In these F1 animals, the maternal allele of *Peg3* is silenced due to the imprinting-driven DNA methylation on the Peg3-DMR. On the other hand, the paternal transmission of the targeted allele is designed to truncate the transcription of *Peg3* through the two poly(A) signals that have been included as part of the inserted cassette (**Fig. 1A**). To further confirm this predicted truncation, we performed RT-PCR analyses using total RNA derived from F1 with paternal transmission of the targeted allele (**Fig. 1C**). The results indicated almost complete truncation of *Peg3* transcription. This was further confirmed with western blotting, showing no detectible levels of the PEG3 protein in the F1 mice (**Fig. 1D**). Collectively, the results described above confirm the successful generation of a new mutant allele for the *Peg3* locus. In this new mutant allele, the inserted cassette truncates transcription of *Peg3*, resulting in no detectible levels of the PEG3 protein.

### Mutational effects on litter sizes

**Figure 2:**
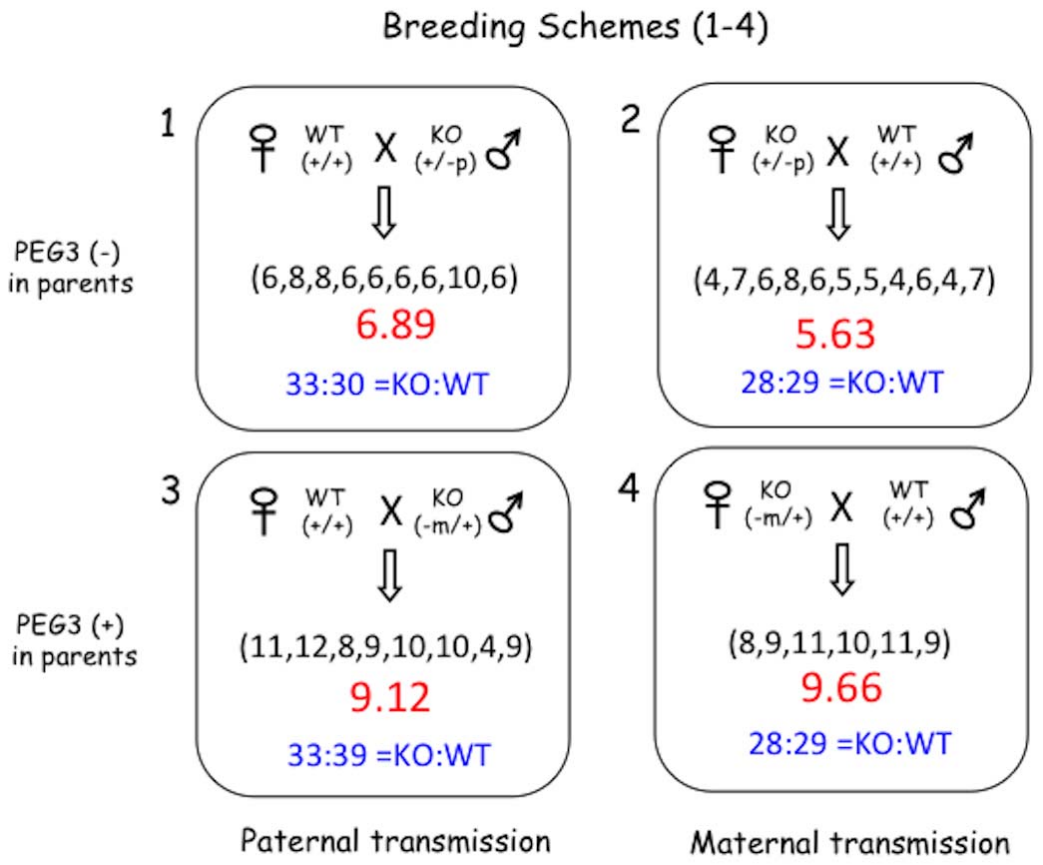
Mutational effects on the litter size of the mouse. Two different types of heterozygotes were used for breeding experiments: males and females inheriting the mutant allele paternally (+/−^P^) versus maternally (−^m^/+). Due to genomic imprinting, the gene dosage of *Peg3* is almost null in the first type (Breeding 1 and 2) whereas the gene dosage in the second type is normal (Breeding 3 and 4). The average litter sizes and the ratios of genotypes (KO versus WT) were shown for the four sets of breeding experiments. The numbers inside parentheses indicate the sizes of individual litters.

With the newly derived mutant line, we performed several series of breeding experiments to characterize potential mutational effects of the targeted allele at the organismal level. Since the *Peg3* locus is imprinted, the heterozygotes can be divided into two types: the first inheriting the targeted allele paternally (+/−^p^) with gene dosage of *Peg3* being null whereas the second inheriting the targeted allele maternally (−^m^/+) with the gene dosage being normal. Thus, this study used four different breeding schemes with the two types of heterozygous males and females (**Fig. 2**). The first set of breeding schemes (1 and 2) bred male and female heterozygotes (+/−^p^) with their wild-type littermates. The second set of breeding schemes (3 and 4) bred male and female heterozygotes (−^m^/+) with their wild-type littermates. The pups derived from these breeding were genotyped, and their body weights were measured and converted into percentile scores as an indicator for their health status. The results derived from these breeding experiments are as follows. The litter sizes (6.89 and 5.63) of the first set of breeding experiments (Breeding 1 and 2) were much smaller than the litter sizes (9.12 and 9.66) of the second set of breeding experiments (Breeding 3 and 4) (**Fig. 2**). Statistical significance of the observed differences are as follows: the p-value is 0.033 for Student t-Test between Breeding 1 versus 3 while the p-value is 0 for Breeding 2 versus 4. Yet, the ratios of heterozygotes to wild-types were all close to the mendelian ratio of 1∶1 among the pups derived from the 4 different breeding schemes. This indicates no lethality associated with the targeted allele during embryonic development. This further suggests that the small litter sizes observed from Breeding 1 and 2 might be caused by unknown mutational effects of the targeted allele on the heterozygous parents (+/−^p^). It is important to note that the gene dosage of *Peg3* is null in these heterozygous parents in contrast to the normal dosage of the heterozygous parents in Breeding 3 and 4.

To further follow up the prediction, we examined the morphology of reproductive organs harvested from the heterozygotes (+/−^p^), and counted the number of germ cells derived from the heterozygotes. There were no major morphological defects in the reproductive organs of male and female heterozygotes except the testes of the heterozygote males were visually smaller than those of the wild-type littermates. The size difference of the testes between the heterozygotes and wild-types was proportional to their body weight differences, suggesting that the smaller-size testes might be an outcome of the overall growth retardation observed in the heterozygotes, which will be described later. On the other hand, the number of mature oocytes found in the heterozygote females (average 5 oocytes per ovary, n = 6) was much smaller than the numbers from the wild-type littermates (average 10 oocytes per ovary, n = 6). This might be consistent with the small litter sizes observed in Breeding 2 with the heterozygous females. In contrast, the number of sperm was highly variable between individual adult mice, thus no statistically significant conclusion could be reached regarding the potential impact of the mutant allele of *Peg3* on the number of sperm. Overall, a series of breeding experiments concluded that the observed small litter sizes are likely caused by Peg3's mutational impact on the reproductive system of the breeding mice, thus suggesting potential roles of *Peg3* in mammalian reproduction.

### Mutational effects on milk provision and growth rates

**Figure 3:**
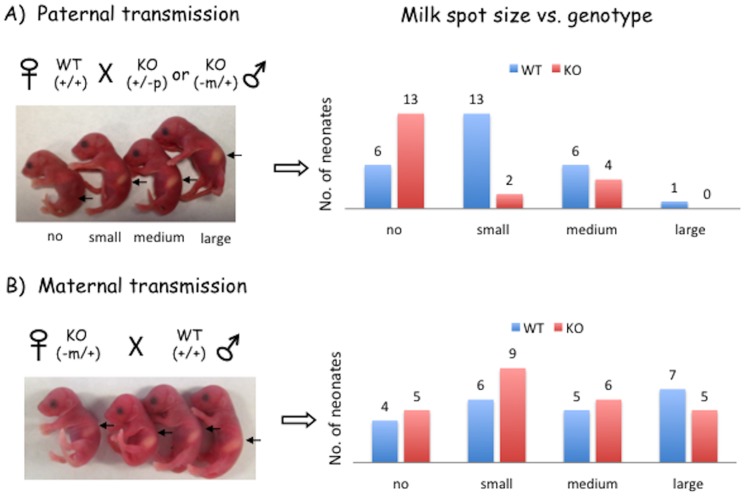
Mutational effects on milk suckling. The pictures on left show one-day-old pups with their stomachs filled with milk (white milk spot). A large fraction of the heterozygotes inheriting the mutant allele paternally (**A**) do not show milk spots as tabulated with a graph on right. On the other hand, the heterozygotes with maternal transmission of the mutant allele (**B**) do not show any correlation between their genotypes and phenotypes (milk spot), since the maternal allele is already silenced by genomic imprinting.

The pups derived from the four breeding schemes were also analyzed to detect potential mutational effects on growth-related phenotypes during early neonatal stages (postnatal day 1 through 7). Monitoring the pups derived two major conclusions. First, half of the heterozygotes (+/−^p^) from Breeding 1 and 3 died during this stage mainly because they were outcompeted for milk by the littermates. To reduce this competition, the litter size from these breeding schemes has been often maintained smaller than 5, which has been an effective approach for securing the survival of the heterozygotes (+/−^p^). In retrospect, this may have been a major reason for high levels of the perinatal lethality observed during the derivation of F1 mice with the germline transmission from the chimeras. To further test this possibility, we scored each pup based on the size of a milk spot on postnatal day 1: no milk spot with an empty stomach, and small, medium and large milk spots (crescent-, half- and full-size oval-shaped stomachs in **Fig. 3**). More than half of the heterozygotes from Breeding 1 and 3 had no milk spot with an empty stomach. In contrast, the pups derived from Breeding 4 did not show any correlation between the genotype and the size of milk spot, confirming that the maternal transmission of the targeted allele has no impact due to the genomic imprinting-driven repression of the targeted allele. Statistical significances of these observations are as follows: the size of milk spot versus the genotype (Χ^2^ test, df = 1, p = 0.0225) and the size of milk spot versus the mode of transmission (Χ^2^ test, df = 1, p = 0.0004). The pups from Breeding 2 were not included for this analysis mainly because their litter size was usually too small, although the entire litter of pups from Breeding 2 often had no milk spot. Overall, these observations indicate that some problems associated with milk suckling are likely the major cause for perinatal lethality observed among the heterozygotes (+/−^p^) derived from the breeding.

**Figure 4:**
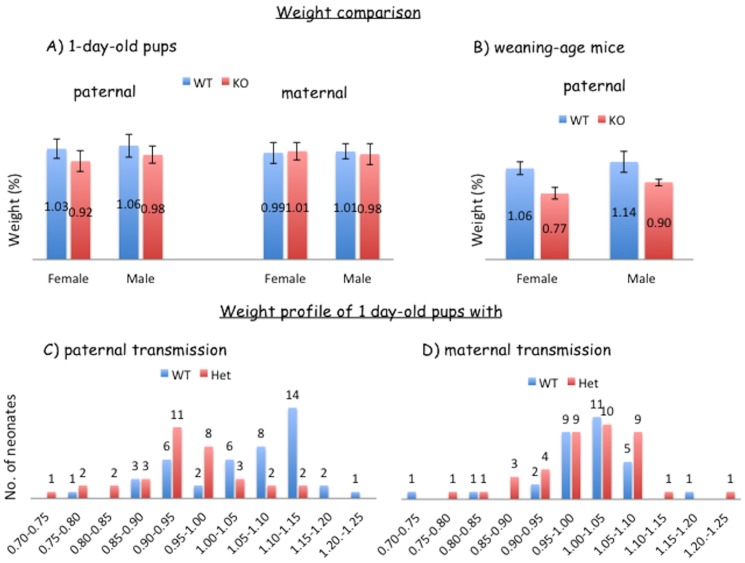
Mutational effects on growth rates. One-day-old pups derived from the four different breeding schemes detailed in Fig. 2 were also analyzed in terms of their birth weight percentiles. Birth weight percentiles were calculated through dividing the weight of each pup by the average weight of the litter. The weight profiles of two genotypes (WT versus KO) were presented for each group either with the paternal (**C**) or maternal (**D**) transmission. The pups inheriting the KO allele paternally showed smaller body weights than their littermates. The values on the X-axis indicate percentile scores, while the values on the Y-axis indicate the number of mice. The overall averages of weight percentiles for these two groups were also compared between the two genders of mice (**A**). The weight profiles of some of these pups were further analyzed similarly at the weaning age (**B**).

Second, besides the observed perinatal lethality, the growth of the heterozygotes with the paternal transmission was overall retarded throughout the neonatal to weaning stages (**Fig. 4**). According to the average weight percentiles of the 1-day-old pups, the heterozygotes (+/−^p^) from Breeding 1 and 3 showed about 10% smaller body weight than their wild-type littermates (Student's t-Test, p = 0.0003 for females and p = 0.01 for males) (**Fig. 4A**). In contrast, the heterozygotes (−^m^/+) from Breeding 2 and 4 did not show any difference compared to those of the wild-type littermates (Student's t-Test, p = 0.53 for females and p = 0.33 for males). The individual pups from these breeding schemes also displayed quite different weight profiles between the paternal and maternal transmission (**Fig. 4C&D**). In the paternal transmission, the peak of weight profile for the heterozygotes is around 90–100%, which is 15% smaller than the peak of the wild-type littermates (105–115%) (**Fig. 4C**). In the maternal transmission, the weight profiles were almost identical between the heterozygotes and wild-type littermates (**Fig. 4D**). We also followed up the initial patterns of growth-related effects through monitoring the weights of weaning-age mice (Postnatal day 21) (**Fig. 4B**). The mice from the maternal transmission did not show any difference between the heterozygotes and wild-type littermates (data not shown). However, the mice from the paternal transmission displayed 24–29% weight difference between the heterozygotes and wild-type littermate in both genders (Student's t-Test, p = 0.0002 for females and p = 0.012 for males). Our continued survey on these adult mice further confirmed that this weight difference between the two genotypes was sustained throughout the lifetime of the mice. Overall, these results indicate that the mutant allele on *Peg3* causes perinatal lethality through unknown problems associated with milk provision. Also, the mutation has a major impact on the growth rates of the mice throughout their lifetime.

### Mutational effects on gene expression in embryo head and placenta

**Figure 5:**
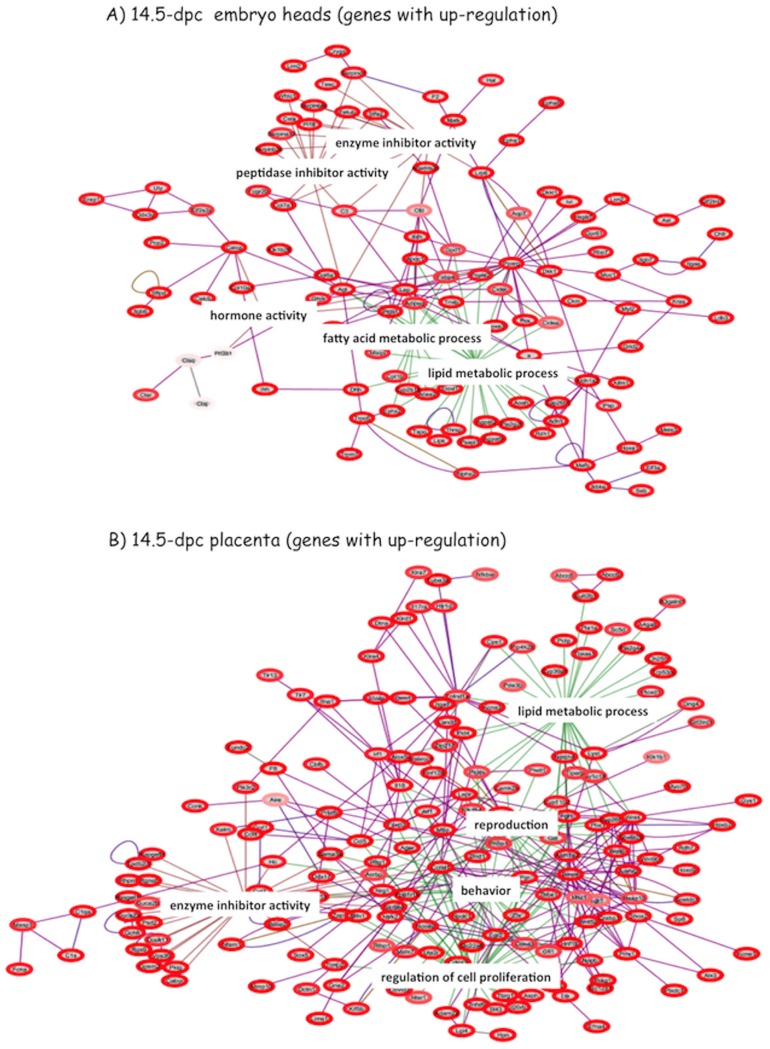
Mutational effects on global gene expression. A litter of 14.5-dpc fetuses were prepared from a crossing of a heterozygote male (−^m^/+) and wild-type female. Global gene expression analyses were performed with a set of total 8 embryo heads and placenta representing one of the following four combinations: male hetetrozygote and wild-type and female heteterozygote and wild-type. This series of expression analyses identified four groups of genes: up- and down-regulated gene sets for embryo head and placenta. These four groups of genes were used for identifying enriched pathways using the bioinformatics program EGAN (Explorative Gene Association Networks). Two of these four results are shown: enriched pathways with up-regulated genes at 14.5-dpc embryo heads (**A**) and placenta (**B**). The remaining two results showing enriched pathways with down-regulated genes at both samples are shown in Fig. S1. The annotation for the diagrams is as follow. Lines: Dark Purple, Human protein-protein interactions; Light Purple, PubMed co-occurrence (literature); Gold, All protein-protein interactions; Grey, Chromosomal sequence (proximity); Red, GO Function, transcription regulator activity; Green, GO Process, generation of neurons. Circles: Red (color gradient), up-regulated genes; darker red signifying higher WT∶KO expression ratio.

We decided to perform genome-wide expression analyses to further understand the observed mutational effects described above. For this series of experiments, one litter of 14.5-dpc (days post coitum) fetuses were obtained through timed mating between a male heterozygote (−^m^/+) and wild-type female littermate. The gender and genotype of each fetus were first determined with PCR using DNA isolated from the amnionic sac. Since *Peg3* is highly expressed in brain and placenta, total RNA was isolated from embryo heads and placenta. This series of experiments analyzed individually each total RNA that had been isolated from 8 sample sets of embryo heads and placenta, representing 4 heterozygotes (+/−^p^) and wild-types, including 4 males and females. The results derived from this series of experiments are as follows. First, the expression levels of a large number of genes are affected in the embryo heads of the heterozygotes: 325 genes displayed up-regulation (>1.5-fold) whereas 162 genes showed down-regulation (<1.5-fold). In placenta, the expression levels of a larger number of genes are also affected: 489 and 378 genes show up- and down-regulation, respectively (**Material S1&S2**). Second, gene-clustering analyses using the affected genes indicate that many genes of the affected genes can be grouped into several biological pathways (**Fig. 5** **, Fig. S1, Material S2**). Among the various pathways, two pathways are the most frequently observed in this series of clustering analyses, lipid metabolism and fatty acid processes (**Fig. 5**). This is also consistent with the previous observation that *Peg3* plays significant roles in adipocyte differentiation and control of growth rates [7].

**Figure 6:**
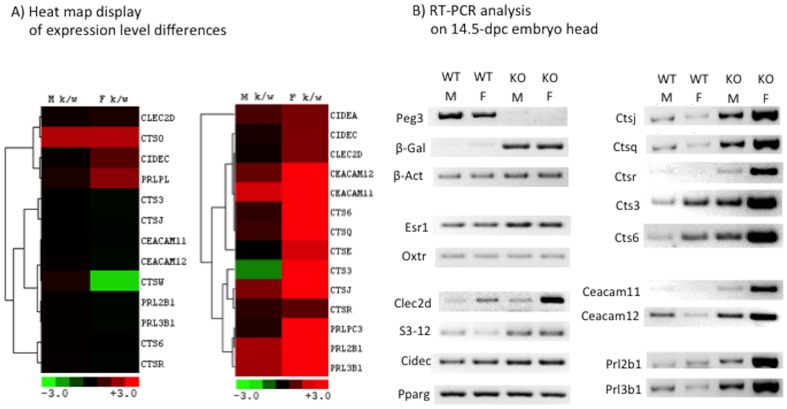
Mutational effects on placenta-specific gene families. The expression levels of three placenta-specific gene families (*Cts*, *Prl*, *Ceacam*) were not affected in placenta (left heat map), but up-regulated in the embryo head of the heterozygotes (right heat map)(**A**). The observed up-regulation of these gene families was further analyzed with RT-PCR using total RNA isolated from the 14.5-dpc embryo heads of the wild-type (WT) and heterozygote embryos (KO) with two different sexes, male (M), female (F)(**B**). The name of each gene analyzed is indicated on the left side of the gel image showing the PCR products.

According to manual inspection of the list of affected genes, the expression levels of two groups of genes were most affected in the embryo head by the mutation. One group includes many genes that are involved in lipid metabolism, such as *Clec2d* (C-type lectin domain family 2 member D), *S3–12*, *Cidea* (cell death activator CIDE-A), *Cidec* (cell death activator CIDE-C) and *Pparg* (peroxisome proliferator-activated receptor gamma). This is again consistent with the fact that *Peg3* is involved in adipocyte differentiation. The other group includes members of placenta-specific gene families, such as prolactin (*Prl3b1* and *Prl2b1*), cathepsin (*Ctsj* and *Ctsq*) and carcinoembryonic antigen cell adhesion molecule (*Ceacam 11* and *12*). The observed changes in the expression levels of these gene families were always up-regulation. Interestingly, the levels of up-regulation were much greater in females (30-fold) than in males (5-fold) (**Fig. 6A**). The changes in these families were more readily detectible since these gene families are not normally expressed at all in the embryo head. In placenta, on the other hand, there was no major difference in the expression levels of these gene families between the wild-types and heterozygotes (**Fig. 6A**). In conclusion, a large number of genes are affected in the mutant mice in terms of their expression levels. Among the affected genes, the most dramatic changes were observed in several placenta-specific gene families, suggesting that *Peg3* might be involved in the transcriptional control of these gene families.

### Mutational effects on the transcription of placenta-specific gene families

We further analyzed the up-regulation of several placenta-specific gene families observed in the embryo heads of the heterozygotes using the total RNA isolated from different-stage brains, 14.5-dpc embryos, neonates and adults. For this series of analyses, total RNA was isolated from the brains of the wild-types and heterozygotes (+/−^p^) with two different genders (**Fig. 6B**). According to the results from independent RT-PCR analyses, the members of the three placenta-specific gene families are indeed up-regulated in the embryo head, consistent with the initial results derived from genome-wide expression analyses (**Fig. 6A**). The confirmed up-regulation was also much higher in females than in males. We also analyzed the total RNA isolated from neonate brains (**Fig. S2**). Two genes (*Ctsj* and *Prl3b1*) were up-regulated in the brains of 1-day-old heterozygotes. At this stage, however, the up-regulation was not obvious in males, but was easily detectible in females, displaying 15- and 30-fold up-regulation for *Ctsj* and *Prl3b1*, respectively. We also performed RT-PCR analyses using the total RNA isolated from the pituitary glands of 6-month-old female mice. This series of analyses also confirmed the up-regulation of *Ctsj* and *Ctsq* in the heterozygotes (**Fig. S2**). However, the up-regulation of the other gene families, *Prl* and *Ceacam*, could not be determined due to the overall low expression levels. Besides these gene families, we also analyzed the expression levels of several other genes, including *Clec2d*, *S3–12*, *Cidec*, *Pparg*, *Esr1* (estrogen receptor alpha) and *Oxtr* (oxytocin receptor) (**Fig. 6B**). Among these genes, the up-regulation was further confirmed for the three genes (*Clec2d*, *S3–12* and *Cidec*), which are involved in lipid metabolism. In conclusion, this series of expression analyses confirmed the up-regulation of placenta-specific gene families in different-stage mouse brains. Since these gene families are expressed mainly in placenta during the gestation period, the observed up-regulation is thought to be an outcome of global de-repression of these gene families in the brains of neonatal and adult mice.

## Discussion

In the current study, a new mutant mouse model has been generated to further characterize the *in vivo* functions of the imprinted gene *Peg3*. The results from breeding experiments indicated potential roles for *Peg3* in mammalian reproduction: females heterozygous for the mutant allele produce a much smaller number of mature oocytes, resulting in reduced litter sizes compared to those of wild-type littermates. The results also revealed partial neonatal lethality among the heterozygotes (+/−^p^) mainly because the heterozygotes are outcompeted for milk by the wild-type littermates. Genome-wide expression analyses indicated the two types of genes most affected by the *Peg3* mutation: the first group being those involved in lipid metabolism and the second group being the members of placenta-specific gene families (prolactin, cathepsin, ceacam). Detailed analyses further indicated that the observed up-regulation of these gene families might be an outcome of de-repression of these placenta-specific gene families in the brain of the *Peg3* mutant mice. Overall, these results suggest that *Peg3* may be involved in transcriptional regulation of these placenta-specific gene families.

The results from breeding experiments clearly indicate potential roles for *Peg3* in mammalian reproduction (**Fig. 2**). The heterozygotes with the paternally transmitted mutant allele (Breeding 1 and 2) tend to have much smaller litter sizes than those with the maternally transmitted mutant allele (Breeding 3 and 4). Yet, the genotypes of the offspring from these 4 breeding schemes are all close to the mendelian ratio of 1 to 1 between the mutant allele carriers and wild-types, suggesting no embryonic lethality associated with these breeding experiments. The two types of heterozygotes used for these breeding experiments are quite different from each other in terms of Peg3's gene dosage due to genomic imprinting (**Fig. 2**). The gene dosage of *Peg3* is almost null in the heterozygotes with paternal transmission (+/−^P^) whereas the dosage is normal in those with maternal transmission (−^m^/+). It is also important to note that mammalian *Peg3* is highly expressed in the two reproductive organs, testis and ovary, suggesting major roles in reproduction [3]. Thus, the small litter sizes may be caused by some unknown defects in the reproductive system of the heterozygotes with paternal transmission. Consistent with this prediction, female heterozygotes (+/−^P^) produce much smaller numbers of mature oocytes than the wild-type littermates. It is not obvious at the moment how the mutation on *Peg3* results in reduction in the number of mature oocytes since *Peg3* function has not been well studied in the mammalian reproductive system. This is also the case for the other imprinted genes, although genomic imprinting is closely tied to mammalian reproduction [28]. Thus, it would be of great interest to further investigate potential roles of *Peg3* in the mammalian reproductive system in the near future.

According to the results from genome-wide expression analyses, the expression levels of many genes are affected in embryo heads and placenta by the *Peg3* mutation (**Fig. 5** **&** **6**). Among the many affected genes, the mutational effects on three placenta-specific gene families are unique based on the following reasons. First, the mutational effects on these gene families are all up-regulation. These gene families are expressed mainly in placenta, but not in embryos [29],[30]. Yet, these gene families are all up-regulated in the brains of the mutant embryos. Second, the mutational effects appear to be global. Each of these gene families occupies relatively large genomic intervals, size-ranging from 0.5 to 2 Mb genomic distance [29]. Detailed examination indicated that the majority of members in each gene family are affected by the *Peg3* mutation (**Fig. 6**). Third, the mutational effects are also somewhat permanent, not temporary, as the observed up-regulation is detected in embryo heads as well as in neonatal and adult brains (**Fig. S2**). These patterns of up-regulation suggest that some unknown setting for the repression of these gene families might not be properly established in the *Peg3* mutant mice. According to the results from the ENCODE project [31], these gene families are marked with the H3K9me3 modification (trimethylation at lysine 9 of histone 3) in the majority of embryonic and adult tissues, but not in placenta where these gene families are highly expressed. In placenta, these gene families are marked with active histone marks, such as H3K27ac and H3K4me3. The H3K9me3 is a well-known repression signal for many gene families, especially for tissue-specific gene families [32]. Thus, it is reasonable to predict that the observed up-regulation might be an outcome of defects in H3K9me3 or H3K9me3-related epigenetic settings in the *Peg3* mutant mice. It is also interesting to note that *Peg3* encodes a transcriptional repressor with DNA-binding capability [26], but it is unclear whether the repression activity of PEG3 is also mediated through H3K9me3. Nevertheless, the observed up-regulation suggests a potential functional connection of *Peg3* to the epigenetic setting of the three placenta-specific gene families.

Genome-wide expression analyses also revealed a previously uncharacterized aspect of the *Peg3* locus, a sexually biased effect by the *Peg3* mutation. The observed up-regulation of the placenta-specific gene families is much more obvious in females than in males (**Fig. 6**). This sexually biased effect by the *Peg3* mutation appears to be reminiscent of several independent observations associated with the *Peg3* locus. First, according to the results from previous breeding experiments with a mutant strain deleting part of the Peg3-DMR, the deletion affects the survival and growth rates of males more severely than females [9]. Second, nutritional studies also indicate that the expression levels of *Peg3* are more easily affected in males than in females by mal-nutrition during the gestation period [33]. Third, many reported disease cases associated with the human *PEG3* locus also tend to be sexually biased. Hypermethylation on the PEG3-DMR has been detected mainly in female-specific cancers, such as ovarian and breast cancers [15],[17],[18]. Along with the sexually biased mutational effect observed in the current study, these independent observations strongly suggest that the functions of *Peg3* might be closely connected to, or influenced by, mammalian sexual differentiation. *Peg3* is also highly expressed in a known sexually dimorphic part of brain, the hypothalamus, further supporting the potential connection of *Peg3* function to mammalian sexual differentiation [28]. In conclusion, although these independent observations need to be further investigated more thoroughly, it becomes more obvious that *Peg3* function is intertwined with mammalian sexual differentiation.

In summary, the current study reports the successful derivation of a new mutant model for *Peg3*. The current study also reports three new observations regarding the *in vivo* roles of *Peg3*: defects in reproduction, perinatal lethality, and ectopic expression of placenta-specific gene families in brains. These observations have never been reported previously, thus providing new insights regarding the *in vivo* roles of *Peg3*. We further highlighted the significance of these observations by comparing with the previous observations that have been derived from another mutant model of *Peg3* (**Table 1**). There are some similarities and differences between the two mutant models. Peg3's roles in controlling milk provision and growth rates are reproducible. In contrast, Peg3's roles in reproduction have not been noticed earlier from another mutant model, which may need further investigation in the near future. Overall, the new mutant model described in the current study should be very useful for further investigating *the in vivo* roles of *Peg3*.

**Table 1:** Comparison of phenotypes derived from the two different mutant models of *Peg3*.

	Previous studies (Ref. No. 5 & 6)	Current study
1. Mutagenesis method	Targeted knockin at Exon 5 followed by transcription truncation	Targeted knockin at Intron 5 followed by transcription truncation
2. Genetic background	129sv	C57BL6/N
3. Mutational effect on litter sizes	1) Paternal transmission Control∶Mutant = 6.86∶5.96	1) Paternal transmission Control∶Mutant = 9.12∶6.89
	2) Maternal transmission Control∶Mutant = 6.86∶6.46	2) Maternal transmission Control∶Mutant = 9.66∶5.63
4. Mutational effect on reproduction	Not available.	Female Peg3^+/−^ heterozygotes have a reduced number of mature oocytes.
5. Mutational effects on lethality	Not available.	Perinatal lethal for Peg3^+/−^ pups - half of the mutant pups die during the Postnatal day 1 through 3.
6. Mutational effects on milk provision and growth rates	Female Peg3^+/−^ heterozygotes have defects in nurturing and milk letdown.	Heterozygous Peg3^+/−^ pups have a milk-suckling problem.
7. Mutational effects at the molecular and cellular levels	Female Peg3^+/−^ heterozygotes have a reduced number of oxytocin neurons.	Female Peg3^+/−^ heterozygotes have ectopic expression of placenta-specific gene families in brains.

## Materials and Methods

### Ethics Statement

All the experiments related to mice were performed in accordance with National Institutes of Health guidelines for care and use of animals, and also approved by the Louisiana State University Institutional Animal Care and Use Committee (IACUC), protocol #10-071.

### Generation of a new mutant allele for *Peg3*

A targeted ES cell (Peg3^tm1aEUCOMMhmgu^) was obtained from EUCOMM, and the DNA isolated from this ES cell was further analyzed using a long-distance PCR scheme with the following primer set: GF3 (5′- GCCAATATCCAACTCATGCTACGTTC-3′) and LAR3 (5′-CAACGGGTTCTTCTGTTAGTCC-3′); RAF5 (5′-CACACCTCCCCCTGAACCTGAAAC-3′) and GR3 (5′- GTTGTTGCTGGTAGTTGAGCGTTGTC-3′). After the confirmation, this ES cell was microinjected into the blastocyst of e3.5-embryos of C57BL/6J (albino mice with the c/c genotype). Among the five chimeras obtained from the injection, two were successfully bred with female breeders of the C57BL/6N origin (black mice with the C/C genotype), producing 6 live F1 with the germline transmission of the targeted allele. These 6 F1 have been used for establishing the mutant line.

### Mouse breeding

Two different types of heterozygotes were prepared for breeding experiment: male and female heterozygotes carrying the paternally (+/−^p^) and maternally (−^m^/+) transmitted targeted allele. These 4 heterozygotes were bred with their littermates: for Breeding 1, female wild-type littermates×male heterozygotes (+/−^P^); Breeding 2, female heterozygotes (+/−^P^)×male wild-type littermates; Breeding 3, female wild-type littermates×male heterozygotes (−^m^/+); for Breeding 4, female heterozygotes (−^m^/+)×male wild-type littermates. One-day-old pups derived from these breeding were genotyped using the following primer set: Peg3-5arm (5′-CCCTCAGCAGAGCTGTTTCCTGCC-3′) and LAR3 (5′-CAACGGGTTCTTCTGTTAGTCC-3′). The genders of these pups were determined using the following primer set: mSry-F (5-GTCCCGTGGTGAGAGGCACAAG-3) and mSry-R (5-GCAGCTCTACTCCAGTCTTGCC-3). The body weights of the pups were also measured as an indicator for their health status. For genotyping and gender determination, genomic DNA was isolated from either clipped tails or ears by incubating the tissues overnight at 55°C in the lysis buffer (0.1 M Tris-Cl, pH 8.8, 5 mM EDTA, pH 8.0, 0.2% SDS, 0.2 M NaCl, 20 µg/ml Proteinase K).

### Genome-wide expression analysis

A litter of 14.5-dpc fetuses were harvested from a timed mating between a male heterozygote (−^m^/+) and wild-type female littermate. Three tissues were obtained from each fetus: embryo head, placenta and amnionic sac. The DNA from amnionic sac was used for determining the genotype and gender of each fetus. Embryo heads and placenta were used for isolating total RNA. Total 16 samples were used for this series of genome-wide expression analyses: 8 embryo heads and placenta representing two biological replicates of each of the four following combinations: male wild-type, male heterozygote, female wild-type, and female heterozygote. The isolated total RNA from each sample was further treated with DNAse I and later purified with columns to remove any genomic contaminations (Qiagen, RNeasy Mini-kit). Each total RNA (2 µg) was used for generating labeled cRNA for hybridization on a mouse expression analysis platform (Illumina, Microarray-Mouse Ref-8 v2 expression beadchip). In brief, each cRNA sample (750 ng) was hybridized onto the expression bead chips at 58°C overnight. Chips were scanned with the Illumina Bead Array Reader (Factor = 1, PMT 520, Filter = 100%), and the numeric results were extracted with GenomeStudio using the Gene Expression Module v.1.0.6. Raw data were background-subtracted and normalized using the quantile normalization method (lumi software package) [34],[35]. Normalized data were filtered for genes with significant expression levels compared to negative control beads. Selection for differentially expressed genes was performed on the basis of arbitrary thresholds for fold changes plus statistical significance according to the Illumina t-test error model (limma software) [36]. The mRNA array data in MIAME compliant has been submitted to the NCBI Gene Expression Omnibus (GEO) database (Accession No. GSE50818). Pathway analyses were also performed with the EGAN (Explorative Gene Association Networks) package with the obtained gene sets with up- and down-regulation [37].

### Quantitative RT-PCR analyses

Total RNA was isolated from the brains of neonates and adults using a commercial kit (Trizol, Invitrogen). The isolated RNA was first reverse-transcribed using the SuperScript III First-Strand Synthesis System (Invitrogen), and the subsequent cDNA was used as a template for quantitative real-time PCR. This analysis was performed with the iQ SYBR green supermix (Bio-Rad) using the iCycler iQTM multicolor real-time detection system (Bio-Rad). All qRT-PCR reactions were carried out for 40 cycles under standard PCR conditions. The results derived from qRT-PCR were further analyzed as described previously [11]. The information regarding individual primer sequences and PCR conditions is also available (**Material S3**).

### Western blot

Neonatal mouse brain tissues were isolated and homogenized in cooled T-PER Protein Extraction Reagent (Pierce) with Proteinase Inhibitor Cocktail Set I (Calbiochem) added in accordance with manufacturer's protocol. Cellular debris was removed by centrifugation for 10 minutes. Protein concentrations were determined by the Bradford assay kit (Thermo Scientific). 6× Loading dye was then added and fifty micrograms of lysate were separated on 10% SDS–PAGE gels then transferred to PVDF membranes (Hybond-P, Amersham) using a Mini Trans-Blot transfer cell (Bio-Rad). Membranes were blocked for 1 hour in the Tris-buffered saline with 0.05% Tween-20 (TBS-T) containing 1% BLOT Quick Blocker Reagent (Calbiochem), and incubated at 4°C overnight with custom-made anti-PEG3 primary antibody [12]. Membranes were then washed multiple times at room temperature in TBS-T and incubated for an additional 1 hour with anti-rabbit secondary antibody linked to horseradish peroxidase (Sigma). Blots were washed and exposed to ECL (Thermo Scientific) and developed against x-ray film.

## Supporting Information

Figure S1 The two groups of gene that are down-regulated at 14.5-dpc embryo heads (**A**) and placentas (**B**) were analyzed to derive enriched biological pathways using the EGAN program.
(TIFF)

Figure S2 The observed up-regulation of the placenta-specific gene families was further confirmed with qRT-PCR using total RNA isolated from 1-day-old brains (**A**) and also pituitary glands of 6-month-old females (**B**).
(TIFF)

Material S1 Lists of genes that are up- and down-regulated at 14.5-dpc embryo heads and placenta by the *Peg3* mutation.
(XLSX)

Material S2 Lists of biological pathways in which the up- and down-regulated genes by the *Peg3* mutation are clustered.
(XLSX)

Material S3 List of primers that were used for qRT-PCR.
(XLSX)
