# Correction: *Peg3* Mutational Effects on Reproduction and Placenta-Specific Gene Families

**DOI:** 10.1371/annotation/8de03ec5-e62c-4135-87ee-4928475090a8

**Published:** 2014-01-16

**Authors:** Joomyeong Kim, Wesley D. Frey, Hongzhi He, Hana Kim, Muhammad B. Ekram, Arundhati Bakshi, Mohammad Faisal, Bambarendage P. U. Perera, An Ye, Ryoichi Teruyama

In the Funding statement, the number of a grant from the National Institutes of Heatlh to the first author (JK) is incorrect. The Funding statement should read: 

"This research was supported by National Institutes of Health (J.K. R01-GM066225, R01097074, and R15-ES019118). The Pennington Biomedical Genomics Core Facility is supported in part by COBRE (NIH P20 GM103528) and NORC (NIH 2P30DK072476) center grants from the National Institutes of Health. The funders had no role in study design, data collection and analysis, decision to publish, or preparation of the manuscript."

