# Supplementary figures and images for: Peg3 Mutational Effects on Reproduction and Placenta-Specific Gene Families
Source: PLoS One. 2013 Dec 31;8(12):e83359. doi: 10.1371/journal.pone.0083359 (PMC3877027; doi:10.1371/journal.pone.0083359)

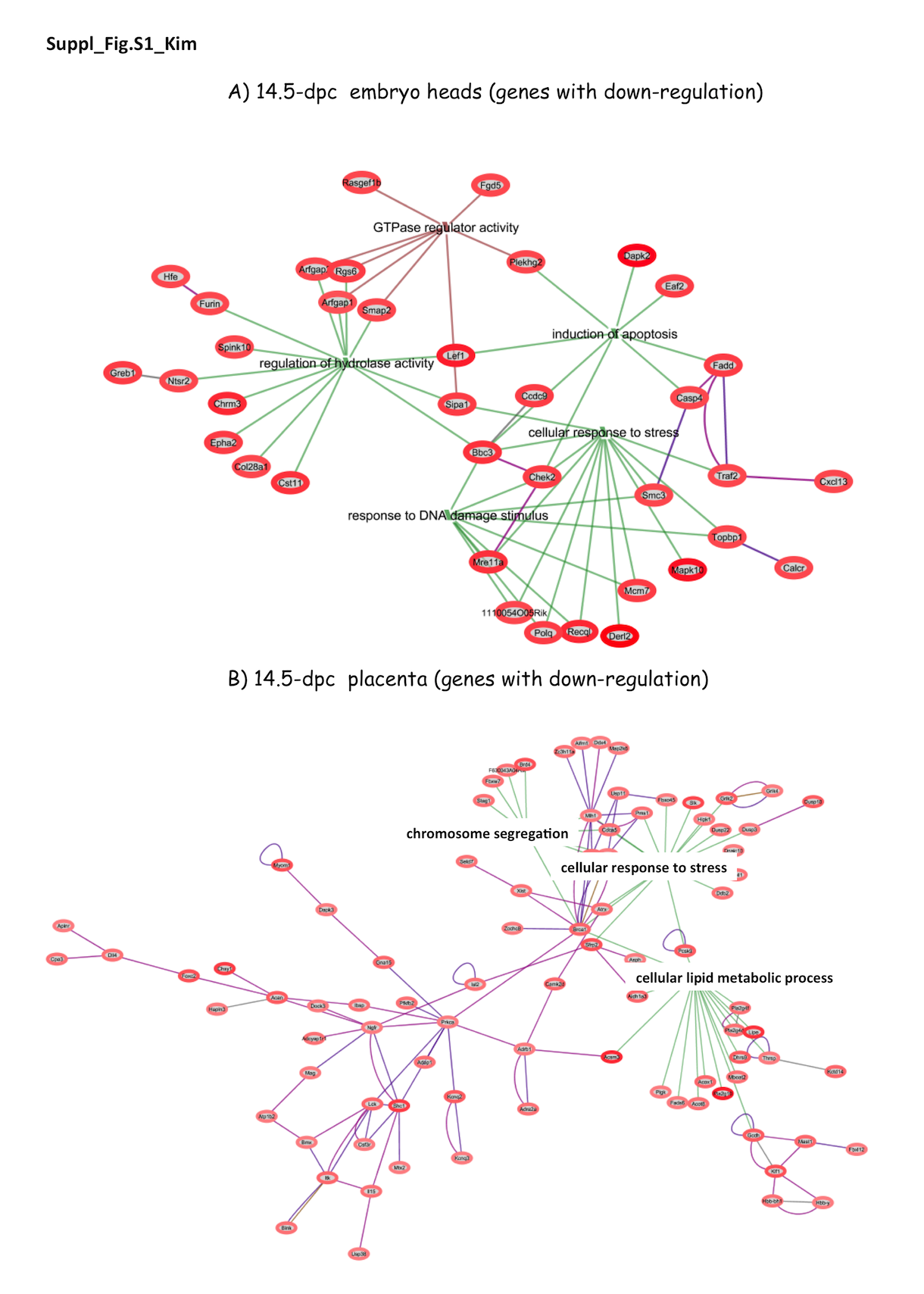

Supplement: Figure S1 — The two groups of gene that are down-regulated at 14.5-dpc embryo heads (A) and placentas (B) were analyzed to derive enriched biological pathways using the EGAN program. (TIFF) [file pone.0083359.s001.tiff]

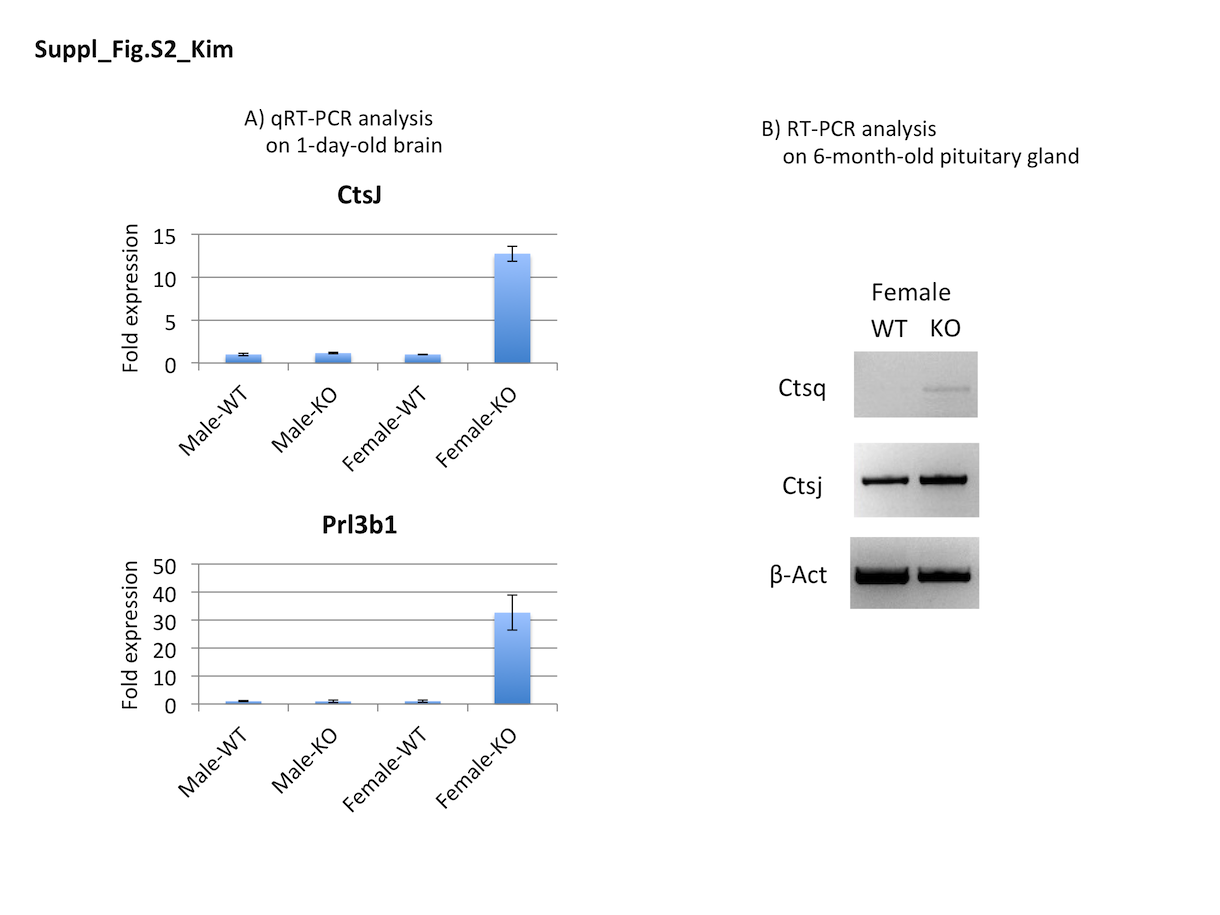

Supplement: Figure S2 — The observed up-regulation of the placenta-specific gene families was further confirmed with qRT-PCR using total RNA isolated from 1-day-old brains (A) and also pituitary glands of 6-month-old females (B). (TIFF) [file pone.0083359.s002.tiff]
